# A simple method for calculation of basic molecular properties of nutrients and their use as a criterion for a healthy diet

**DOI:** 10.12688/f1000research.10537.1

**Published:** 2017-01-05

**Authors:** Veljko Veljkovic, Vladimir Perovic, Marko Anderluh, Slobodan Paessler, Milena Veljkovic, Sanja Glisic, Garth Nicolson

**Affiliations:** 1 Biomed Protection, Galveston, USA; 2 Faculty of Pharmacy, University of Ljubljana, Ljubljana, Slovenia; 3 Department of Pathology, Galveston National Laboratory,, University of Texas Medical Branch, Galveston, USA; 4 Department of Molecular Pathology, The Institute for Molecular Medicine, Huntington Beach, USA

**Keywords:** healthy diet, nutrients, human milk, molecular descriptors

## Abstract

*Background: *Healthy nutrition is vital for good health and well-being. Despite the important role of a healthy nutritional diet, recommendations for healthy eating remain elusive and are mainly based on general properties of nutrients. The present study proposes an improved characterization of the molecular characteristics of nutrients, which are important for biological functions and can be useful in describing a healthy diet. *Methods: *We investigated the electronic properties of some known nutrient ingredients. In this analysis, we used the average quasi valence number (AQVN) and the electron-ion interaction potential (EIIP), which are molecular descriptors that represent the basic electronic properties of organic molecules. *Results: *Our results show that most nutrients can be represented by specific groups of organic compounds according to their basic electronic properties, and these differ from the vast majority of known chemicals. Based on this finding, we have proposed a simple criterion for the selection of food components for healthy nutrition. *Discussion: *Further studies on the electronic properties of nutrients could serve as a basis for better understanding of their biological functions.

## Abbreviations

BCS - basic chemical space

d1 - domain to the left of BCS

d2 - domain to the right of BCS

N(d1) - fraction of nutrients in the d1 domain

N(d2) - fraction of nutrients in the d2 domain

## Introduction

Healthy eating behavior and physical activity patterns promote good mental and physical health and reduce the rates of chronic morbidity and mortality. In the U.S., the Centers for Disease Control and Prevention estimate that in the U.S. alone “poor diet and physical inactivity cause 310,000 to 580,000 deaths per year and are major contributors to disabilities that result from diabetes, osteoporosis, obesity and stroke” ^1^.

The world’s population is aging, and there is a world-wide increase in the prevalence of chronic diseases ( http://www.who.int/ageing/publications/global_health.pdf). The continuing increase in overweight individuals and obesity, which predispose susceptible populations to chronic disease ( http://www.un.org/esa/population/publications/worldageing19502050), emphasize the importance of understanding the impact of nutrition in chronic disease prevention and control.

Optimal nutrition starts with healthy eating habits and assumes a diet that provides the body with essential nutrition, including adequate calories and essential amino acids from proteins, essential fatty acids, vitamins, minerals, and trace nutrients. The crucial part of healthy nutrition is providing a balanced diet, which means consuming foods from all the different nutrient groups (whole grains, fruit and vegetables, diary, protein, fat and sugar) in appropriate quantities. It is also recommended that a healthy nutritional diet favors plant-based foods over animal-based foods. This superficial and elusive definition of a “healthy diet” is often confusing and leads to inappropriate selections of foods. Some animal-based foods should not be selectively avoided, because they contain important nutrients. For example, seafood is an excellent source of the long chain omega-3 fatty acids, and organ meats, such as liver, kidney and heart, as found in beef, sardines, and mackerel, are rich in the coenzyme Q10 and trace nutrients ( https://en.wikipedia.org/wiki/Nutrient). On the other hand, vegetables and fruits contain nutrients with very different biological properties (for example, polyphenols and carotenoids), which are important in a balanced diet.

In addition, increasing protein (“protein diet”) for weight management has become popular, despite some potential adverse effects of this diet, due to carcinogen ingestion of heated or processed meats consumption ^2– 4^. All current recommendations for healthy nutrition suggest avoiding consumption of foods containing high concentrations of saturated fat, due to serious long-term risks of contracting cardiovascular diseases (CVD). However, a recent study on the association between food consumption and CVD, which included data collected from 46 European countries in the period 1980–2008, showed the lack of a connection between saturated fat and CVD. The authors called for serious reconsideration of current dietary recommendations ^5^.

To obtain a better definition of healthy nutrition, it is necessary to know how nutrients execute their biological function. Recently, Norheim *et al*. ^6^ proposed the concept of “molecular nutrition research,” which they defined as “science concerned with the effect of nutrients and foods/food components on whole body physiology and health status at a molecular and cellular level” ^6^.

Previously, we have proposed that the electronic properties of organic molecules represented by the average quasi valence number (AQVN) and the electron-ion interaction potential (EIIP), play an essential role in the determination of their biological properties ^7^. These molecular descriptors, which characterize the long-range molecular interactions (distances between 5 and 1000 Å) in biological systems ^7^, are derived from Mendeleev’s periodic table and determined only by atomic and valence numbers of atoms in a molecule ^8, 9^.

We previously showed that 90.5% of 4,5010,644 compounds randomly selected from the PubChem database ( http://pubchem.ncbi.nlm.nih.gov) have EIIP and AQVN values in the intervals (0.00 – 0.10 Ry) and (2.4 – 3.2), respectively ^10^. This domain of the EIIP/AQVN space, encompassing the majority of known chemical compounds, was referred as the “basic chemical space” (BCS) ^10^. The domains to the left of BCS (d1) and to the right of BCS (d2) encompass 4.3% and 5.3% of analyzed compounds from PubChem, respectively. Compounds located within the domain d1 have strong electron-donor properties and compounds in the domain d2 are strong electron-acceptors ^7^. It was also showed that biological properties of organic molecules (e.g. antibiotics, cytostatics, antiviral compounds, neurotoxins, pheromones, antiparasitic molecules, etc.) are characterized by the electronic properties which are represented by specific domain of the AQVN/EIIP space ^7, 10, 11^. Recently, this finding served as base for development of the criterion for the *in silico* screening of approved drugs for candidate anti-Ebola drugs ^12^. This analysis suggested ibuprofen as a candidate molecule for treatment of the Ebola disease ^13^. The anti-Ebola activity of ibuprofen was later experimentally confirmed ^14^.

Here we present a molecular descriptor analysis of 227 essential and non-essential nutrients and phytonutrients. The comparison of these substances and biologically active compounds in PubChem and ChemBank Databases reveal that food components are characterized by specific electronic properties represented by AQVN and EIIP. Therefore, specific AQN/EIIP molecular descriptors could be regarded as a simple quantitative structure-activity relationship (QSAR) criterion for a selection of food components in healthy diets. Further studies of these molecular descriptors of nutrients, which distinguish them from most other known organic molecules, will help in better understanding their role in essential biological processes.

## Method

### Data sources

The following compounds were assessed by the present study: 227 commonly used organic nutrients ( Dataset 1 ^15^) ( https://en.wikipedia.org/wiki/List_of_micronutrients; https://en.wikipedia.org/wiki/List_of_macronutrients; https://en.wikipedia.org/wiki/List_of_phytochemicals_in_food); 4,667 biologically active compounds from the small bioactive molecule database of ChemBank ( Dataset 2 ^16^) ( http://chembank.broad.harvard.edu); 126 organic nutrients from human milk ( Dataset 3 ^17^) ( http://doublethink.us.com/paala/wp-content/uploads/2012/11/whats-in-breastmilk-poster-canada.jpg), 101 compounds isolated from pomegranate ( Dataset 4 ^18^) ^19^; 42 ingredients of the liquid diet Fresubin ( Dataset 5 ^20^) ( http://www.fresenius-kabi.co.uk/4824_4889.htm).

### Molecular descriptors AQVN and EIIP

Molecular descriptors AQVN and EIIP, determining the long-distance (>5Å) intermolecular interactions in biological systems ^7^, were derived from the “general model pseudopotential” ^8, 9^ and were defined by the following equations:

$$W=0.25\frac{Z^{\text{∗}}\sin\left(1.04\pi Z^{\text{∗}}\right)}{2\pi }\;(\text{E}q.1)$$

where Z* is the AQVN determined by:

$$Z^{\text{∗}}=\frac{1}{N}\sum_{i=1}^{m}n_{i}Z_{i}\;(\text{E}q.2)$$

where *Z _i_* is the valence number of the *i*-th atomic component, *n _i_* is the number of atoms of the *i*-th component, *m* is the number of atomic components in the molecule, and *N* is the total number of atoms. The EIIP values calculated according to equations ( Eq.1) and ( Eq.2) are in Rydbergs (Ry).

## Results

**Figure 1.**
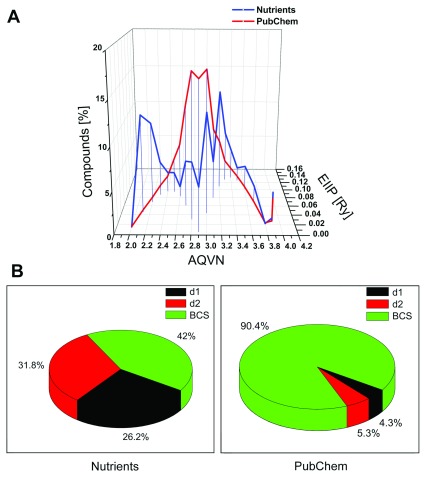
Distribution of nutrients and compounds from PubChem database according their electronic properties represented by the molecular descriptors AQVN and EIIP. ( **A**) Distribution of 227 nutrients (blue) and 4,5010,644 compounds randomly selected from the PubChem database (red) in the AQVN/EIIP space. ( **B**) Percentage of nutrients and compounds from the PubChem database in the AQVN/EIIP domains BCS, d1 and d2. AQVN, average quasi valence number; EIIP, electron-ion interaction potential; BCS, basic chemical space.

The present analysis of 227 essential and non-essential organic nutrients and fitonutrients ( Dataset 1 ^15^) showed significantly different distributions of these compounds in the AQVN/EIIP space compared to compounds from the PubChem database ^10^ ( Figure 1A). Domains d1 and d2 contained 26.2% (N(d1)) and 31.8% (N(d2)) of nutrients, respectively. This result showed that the basic electronic properties defined by AQVN/EIIP of most nutrients (58.2%) significantly differed from the electronic properties of the vast majority of known chemical compounds ( Figure 1B).

**Figure 2.**
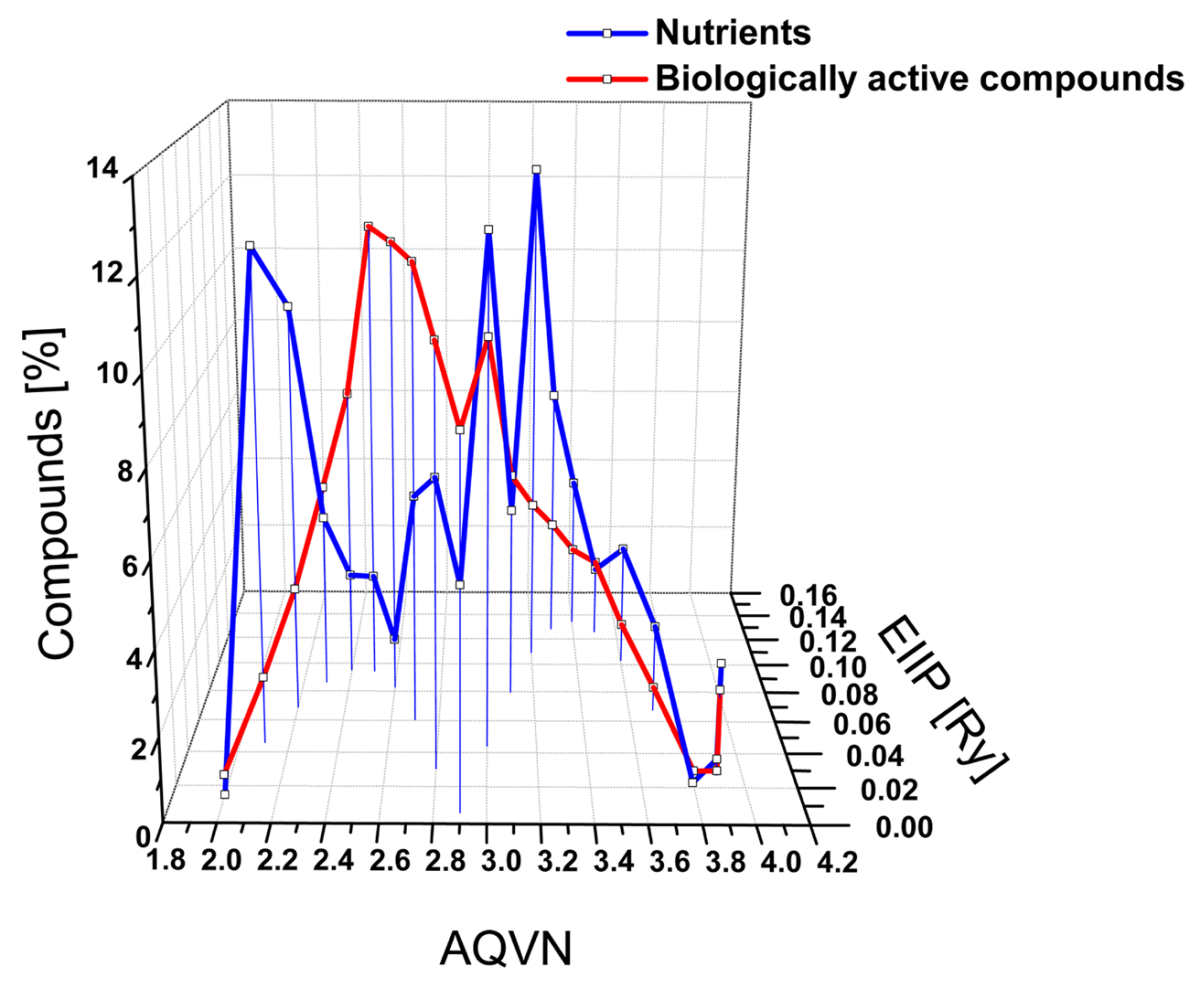
Comparison of distribution of nutrients and naturally occurring biologically active compounds in the AQVN/EIIP space. Distribution of 227 nutrients (blue) and 4667 biologically active compounds (red). AQVN, average quasi valence number; EIIP, electron-ion interaction potential.

To verify that the placement of compounds in domains d1 and d2 were due to specific characteristics of nutrients, we compared them with 4,667 biologically active compounds obtained from ChemBank ( Dataset 2 ^16^). The results presented in Figure 2 show that the distribution of biologically active compounds in the AQVN/EIIP space is similar to distribution of compounds from PubChem ( Figure 1B), but it is different from the allocation of nutrients in this space. The percentage of biologically active compounds that reside outside of the limits of the BCS was also significantly different than the percentage of nutrients (23.9 vs. 58.2%). This confirms that the basic electronic properties of most nutrients also differ from those of other biologically active compounds.

**Figure 3.**
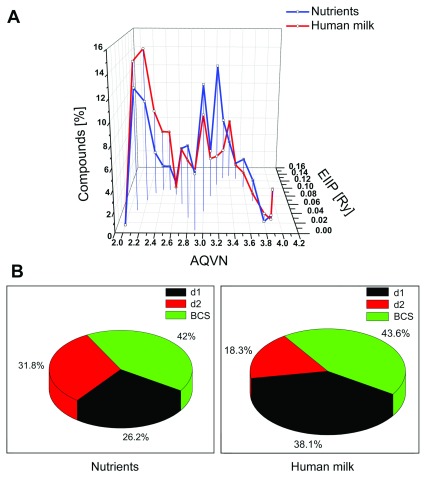
Comparison of distribution in the AQVN/EIIP space of nutrients and ingredients of human milk. ( **A**) Distribution of 227 nutrients (blue) and 126 organic ingredients in human milk (red) in the AQVN/EIIP space. ( **B**) Percentage of nutrients and organic ingredients of human milk in the AQVN/EIIP domains BCS, d1 and d2. AQVN, average quasi valence number; EIIP, electron-ion interaction potential; BCS, basic chemical space.

As an example of a complex natural nutrient fluid, we next analyzed 126 ingredients in human milk ( Dataset 3 ^17^) in order to see how the presence of nutrients with the electronic properties of N(d1) and N(d2) reflected the composition of a complex food that contains all components necessary for development and growth of human tissue. This analysis showed that 56.4% of ingredients of human milk are located outside BCS (38.1% N(d1) and 18.3% N(d2)). Although the general range of distribution of known nutrients in the AQVN/EIIP space ( Dataset 1 ^15^) and the range of distribution of ingredients in human milk ( Dataset 3 ^17^) was similar ( Figure 3A), the percentage content of N(d1) and N(d2) components was different. In contrast to the nutrients that contain similar percentages of N(d1) and N(d2) components, the content of components in human milk was found to be two-times higher in d1 than in the d2 domain ( Figure 3B). Of note is that the content of essential nutrients (essential fatty acids, essential amino acids, vitamins) and non-essential nutrients (unsaturated fatty acids, saturated fatty acids, carbohydrates, non-essential amino acids) presented in Dataset 1 ^15^ is five-times higher in d1 than in the d2 domain (42 vs. 8%). These results suggest that N(d1) components are on average more important for function at the beginning of life, where processes of human organism growth are much more intensive than in the elder organism.

An interesting comparison can be made with Pomegranate ( *Punica granatum*), which has drawn a great deal of attention from both the scientific community and the general public, due to its demonstrated ability to suppress the formation of cancers ^21– 23^ and assist in protection against cardiovascular diseases ^24, 25^ and infections ^26^. Analysis of 101 compounds isolated from pomegranate ( Dataset 4 ^18^) showed that 67% of these compounds are located outside the BCS (26% in d1 and 41% in d2). The extraordinarily high percentage of pomegranate ingredients in the d2, which was significantly higher than the percentage of ingredients of human milk located in this AQVN/EIIP domain, suggests that these ingredients are likely to be essential for the protective effects of this fruit against the development of various diseases.

**Figure 4.**
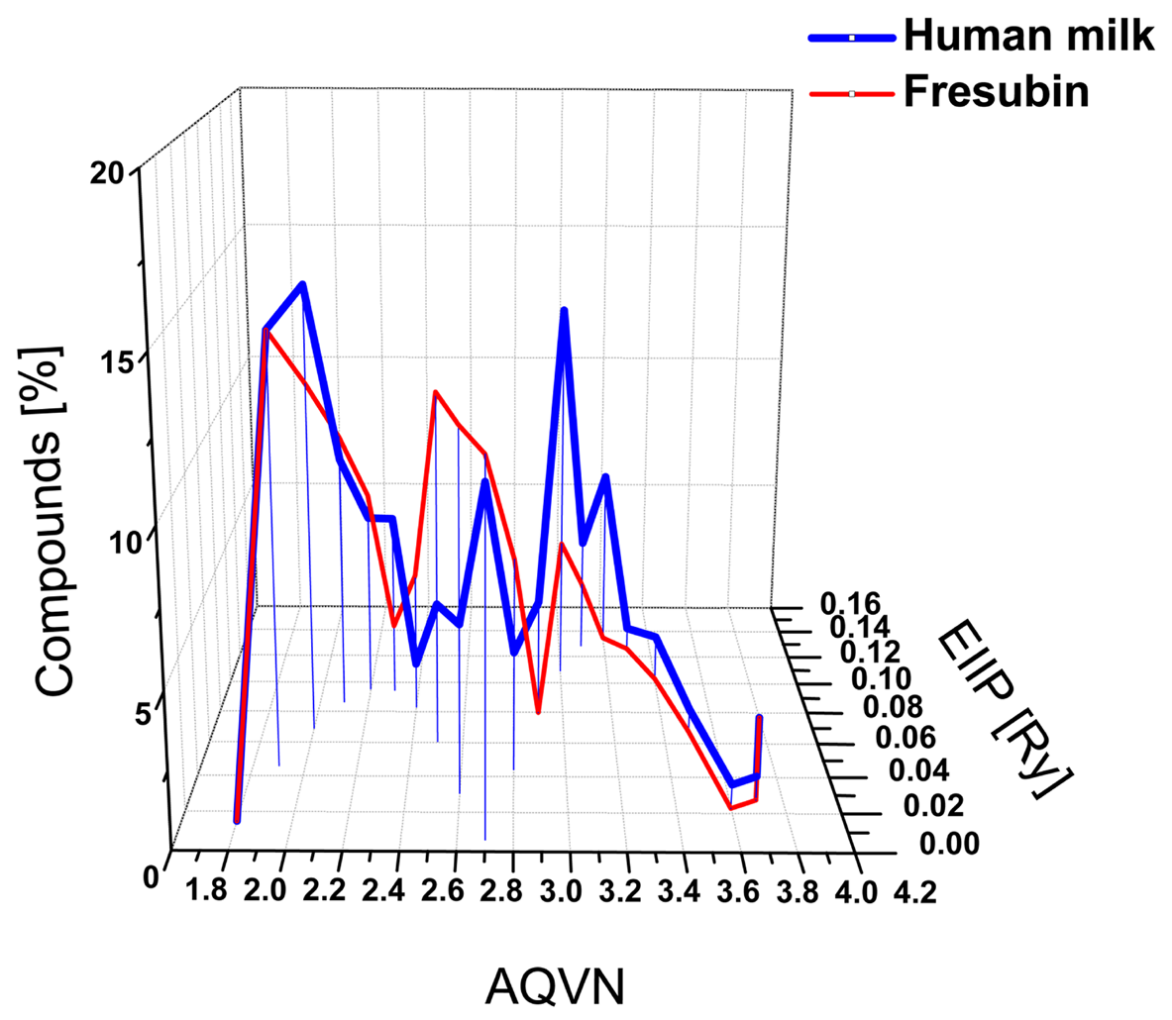
Comparison of distribution in the AQVN/EIIP space of human milk ingredients and organic ingredients of the liquid diet Fresubin. Distribution of 126 organic ingredients of human milk (blue) and 42 organic ingredients of Fresubin (red). AQVN, average quasi valence number; EIIP, electron-ion interaction potential.

Finally, we analyzed the distribution of 42 ingredients ( Dataset 5 ^20^) from the liquid supplement Fresubin, a nutritionally complete liquid diet designed for patients suffering from malnutrition or obstructions of the gastrointestinal tract ( http://www.fresenius-kabi.co.uk/4824_4889.htm). The majority of ingredients (62%) in this liquid nutritional product were distributed outside the BCS. Of these ingredients, 50% were found to be N(d1) and 12% were found to be N(d2). Comparison of the distribution in the AQVN/EIIP space of Fresubin and human milk ingredients is provided in Figure 4.

The AQVN and EIIP of 227 commonly used organic nutrients collected from various sources Click here for additional data file. Copyright: © 2017 Veljkovic V et al. 2017 Data associated with the article are available under the terms of the Creative Commons Zero "No rights reserved" data waiver (CC0 1.0 Public domain dedication).

The AQVN and EIIP of 4667 biologically active compounds from the small bioactive molecule database ChemBank Click here for additional data file. Copyright: © 2017 Veljkovic V et al. 2017 Data associated with the article are available under the terms of the Creative Commons Zero "No rights reserved" data waiver (CC0 1.0 Public domain dedication).

The AQVN and EIIP of 126 organic nutrients from human milk collected from various sources Click here for additional data file. Copyright: © 2017 Veljkovic V et al. 2017 Data associated with the article are available under the terms of the Creative Commons Zero "No rights reserved" data waiver (CC0 1.0 Public domain dedication).

The AQVN and EIIP 42 ingredients of the liquid diet Fresubin Click here for additional data file. Copyright: © 2017 Veljkovic V et al. 2017 Data associated with the article are available under the terms of the Creative Commons Zero "No rights reserved" data waiver (CC0 1.0 Public domain dedication).

The AQVN and EIIP 101 compounds isolated from pomegranate Click here for additional data file. Copyright: © 2017 Veljkovic V et al. 2017 Data associated with the article are available under the terms of the Creative Commons Zero "No rights reserved" data waiver (CC0 1.0 Public domain dedication).

## Discussion

Most nutrients, according to their basic electronic properties, differ from the majority of known chemical compounds, and in the present study we found these preferentially located within the BCS domain. Nutrients outside the BCS domain can be divided in two groups: N(d1) and N(d2) which are located in domains to the left of BCS and to the right of BCS, respectively. We found that in the contents of human milk the number of N(d1) was two-times higher than N(d2). Taking into account the essential role of breast milk in human development, we can speculate that N(d1) is important for the maintenance of basic functions in human organisms, especially in the early phases of intensive growth and development. This assumption was supported by the fact that the N(d1) in a liquid diet designed for chronic illness patients was four times higher than N(d2). As this supplement’s formulation was designed based on empirical rules and is recommended for patients with malnutrition, it is not a coincidence that it resembles milk by composition and is a role model for a balanced diet. More importantly, it offers proof-of concept that the AQVN/EIIP defines food with similar compositions and clearly distinguishes nutrients within a vast chemical space. A high fraction of N(d2) in pomegranate components, which represents a nutritional mixture useful as a supplement in a wide range of human diseases, suggests a protective role of N(d2)-rich compounds. This conclusion is in accord with the fact that polyphenols, which have been confirmed as useful protecting agents against different chronic and infectious diseases ^27– 29^, are largely represented in the d2 domain.

These findings allow the division of foods into two categories, according to their AQVN/EIIP properties: N(d1)- and N(d2)-rich foods. This division serves as a basis for the selection of foodstuffs for a healthy diet with a N(d1)/N(d2) ratio corresponding to human milk and for a diet that is richer in N(d2) and which could have some protective effect against chronic and infectious diseases.

In conclusion, our results demonstrate that most nutrients represent specific groups of organic compounds that can be identified according to their basic electronic properties, which that can be effectively calculated with AQVN/EIIP. In the present study, nutrients were found to differ in their electronic properties from the majority of known chemicals. Additional studies on these properties could help to develop an improved understanding of the role of nutrients in the development and function of human organisms, as well as in protection against various diseases.

## Data availability

The data referenced by this article are under copyright with the following copyright statement: Copyright: © 2017 Veljkovic V et al.

Data associated with the article are available under the terms of the Creative Commons Zero "No rights reserved" data waiver (CC0 1.0 Public domain dedication). 

**Dataset 1**: The AQVN and EIIP of 227 commonly used organic nutrients collected from various sources. DOI, 10.5256/f1000research.10537.d147796 ^15^

**Dataset 2:** The AQVN and EIIP of 4667 biologically active compounds from the small bioactive molecule database ChemBank. DOI, 10.5256/f1000research.10537.d147797 ^16^

**Dataset 3:** The AQVN and EIIP of 126 organic nutrients from human milk collected from various sources. DOI, 10.5256/f1000research.10537.d147798 ^17^

**Dataset 4:** The AQVN and EIIP 42 ingredients of the liquid diet Fresubin. DOI, 10.5256/f1000research.10537.d147799 ^18^

**Dataset 5:** The AQVN and EIIP 101 compounds isolated from pomegranate. DOI, 10.5256/f1000research.10537.d147800 ^20^
