# Supplementary material for: A simple method for calculation of basic molecular properties of nutrients and their use as a criterion for a healthy diet
Source: F1000Res. 2017 Jan 5;6:13. [Version 1] doi: 10.12688/f1000research.10537.1 (PMC5428496; doi:10.12688/f1000research.10537.1)
Supplement: Peer review history

# Peer review: Referee response for version 1

**DOI:** 10.5256/f1000research.11355.r22039

**Published:** 2017-05-08

**Authors:** William W. Stringer

**Affiliations:** 1 David Geffen School of Medicine, University of California, Los Angeles (UCLA), Los Angeles, CA, USA

**Summary**:  Veljkovic et al. have investigated the electronic properties of some known human nutrients and biomolecules. They utilize average quasi valence number (AQVN) and the electron-ion interaction potential (EIIP) as molecular descriptors of the properties of organic molecules (as they have in prior publications).  They were able to represent groups of organic compounds according to their basic electronic properties.  The authors conclude that simple criterion for the selection of food components for healthy nutrition can be identified, and will serve as a basis for better understanding of the nutrients biological functions in the future.

**Major Issues:**

 None

   

**Minor Issues:**

  This reviewer wonders if Fresubin (being an "unflavoured liquid consisting of protein (milk and soya), fat (soya, MCT, linseed, sunflower and fish oils), carbohydrate (maltodextrin), vitamins, minerals and trace elements.") and human milk compare so favorably (Figure 4) due to similar starting materials (milk proteins, minerals, and trace elements)?

I have read this submission. I believe that I have an appropriate level of expertise to confirm that it is of an acceptable scientific standard.

# Peer review: Referee response for version 1

**DOI:** 10.5256/f1000research.11355.r19213

**Published:** 2017-01-30

**Authors:** Timothy K Roberts

**Affiliations:** 1 Tom Farrell Institute for the Environment, University of Newcastle, Callaghan, NSW, Australia

This paper represents a further publication of a novel approach to defining functional properties of biological molecules.  Previously the authors have proposed that the average quasi valence number (AQVN) and the electron-ion interaction potential (EIIP) play a determining role in the biological function of an organic molecule, presumably through facilitating molecular interactions.  Calculations of these values are based on the atomic and valence numbers of atoms in the molecule in question.  This paper addresses the question of consistency in AQVN and EIIP values in relation to known nutritional function. The data support the conclusion that known nutritionally important substances conform to definite subgroupings of AQVN and EIIP values.

 The paper raises many questions in the mind of a traditional biochemist not the least of which is how does this form of analysis relate to the protein components of milk for example?  It is well known that the nutritional and biological function of milk is not only dependent on the small molecules but is also dependent on the protein growth factors present as well.

I have read this submission. I believe that I have an appropriate level of expertise to confirm that it is of an acceptable scientific standard.
